# The Therapeutic Potential of Non-Invasive and Invasive Cerebellar Stimulation Techniques in Hereditary Ataxias

**DOI:** 10.3390/cells12081193

**Published:** 2023-04-20

**Authors:** Alberto Benussi, Giorgi Batsikadze, Carina França, Rubens G. Cury, Roderick P. P. W. M. Maas

**Affiliations:** 1 Neurology Unit, Department of Clinical and Experimental Sciences, University of Brescia, 25121 Brescia, Italy; 2 Department of Neurology and Center for Translational Neuro- and Behavioral Sciences (C-TNBS), Essen University Hospital, University of Duisburg-Essen, 45147 Essen, Germany; 3 Movement Disorders Center, Department of Neurology, University of São Paulo, São Paulo 05508-010, Brazil; 4 Department of Neurology, Donders Institute for Brain, Cognition, and Behaviour, Radboud University Medical Center, 6500 HB Nijmegen, The Netherlands

**Keywords:** degenerative cerebellar ataxias, non-invasive brain stimulation, transcranial direct current stimulation, transcranial magnetic stimulation, dentate nucleus deep brain stimulation, cellular mechanisms, network effects, cerebellar brain inhibition, cerebellum

## Abstract

The degenerative ataxias comprise a heterogeneous group of inherited and acquired disorders that are characterized by a progressive cerebellar syndrome, frequently in combination with one or more extracerebellar signs. Specific disease-modifying interventions are currently not available for many of these rare conditions, which underscores the necessity of finding effective symptomatic therapies. During the past five to ten years, an increasing number of randomized controlled trials have been conducted examining the potential of different non-invasive brain stimulation techniques to induce symptomatic improvement. In addition, a few smaller studies have explored deep brain stimulation (DBS) of the dentate nucleus as an invasive means to directly modulate cerebellar output, thereby aiming to alleviate ataxia severity. In this paper, we comprehensively review the clinical and neurophysiological effects of transcranial direct current stimulation (tDCS), repetitive transcranial magnetic stimulation (rTMS), and dentate nucleus DBS in patients with hereditary ataxias, as well as the presumed underlying mechanisms at the cellular and network level and perspectives for future research.

## 1. Introduction

Cerebellar ataxias encompass a diverse group of hereditary and acquired diseases with various clinical presentations, including loss of balance, uncoordinated limb movements, oculomotor disorders, and slurred speech [1]. Non-motor symptoms, such as cognitive decline and mood disorders, frequently occur in parallel when adequately examined or asked for, but often remain underrecognized [2]. These usually include impairments in one or more of the following domains: executive functions, visuospatial cognition, linguistic processing, and affect regulation [3]. Ataxias are relatively common conditions, with an estimated global prevalence rate of 26/100,000 in children and a global prevalence rate of hereditary cerebellar ataxias of 5/100,000 [4].

Many genetic ataxias are referred to as spinocerebellar ataxias (SCAs), indicating simultaneous or sequential degeneration of the cerebellum and spinal cord as the defining features. However, in many SCAs there are no overt clinical signs of spinal cord involvement, and additional central and peripheral nervous system structures, such as the basal ganglia and peripheral nerves, may well be affected [5]. Several autosomal recessive and X-linked ataxias are also referred to as SCAs, with the addition of an R or X, respectively [6,7]. There are a large number of other genetic forms of ataxia, such as dentatorubral-pallidoluysian atrophy [8], episodic ataxias [9], Friedreich ataxia (FRDA) [10], ataxia-telangiectasia [11], ataxia with oculomotor apraxia (AOA) types 1, 2, and 4 [12,13], hereditary vitamin E deficiency [14], cerebellar ataxia neuropathy and vestibular areflexia syndrome (CANVAS) [15], cerebrotendinous xanthomatosis [16], fragile X-associated tremor/ataxia syndrome (FXTAS) [17], and mitochondrial ataxias [18].

The vast majority of degenerative ataxias currently lack effective disease-modifying therapies, which underscores the growing interest in finding novel innovative strategies to decrease the severity of patients’ symptoms. Five years ago, the Guideline Development, Dissemination, and Implementation Subcommittee of the American Academy of Neurology systematically reviewed the evidence regarding ataxia treatment, concluding that only a few studies showed truly beneficial effects of drugs and physical therapy in a small subset of etiologies [19]. Notably, the development of effective pharmacological treatments aimed at symptomatic improvement is hampered by the pathophysiological heterogeneity of ataxias, and specific therapeutic approaches may be required for each disease.

Cerebellar transcranial direct current stimulation (tDCS) and transcranial magnetic stimulation (TMS) have recently gained increasing attention from the ataxia community because they are non-invasive, may induce neural plasticity irrespective of the underlying disease [20,21,22,23,24,25,26], provide novel information on (patho)physiology, and can be tailored to the needs of individual patients. In addition, several smaller studies have examined dentate nucleus deep brain stimulation (DBS) as an invasive means to directly modulate cerebellar output. In this paper, we comprehensively review the literature on these three stimulation techniques in hereditary ataxias, including the presumed underlying mechanisms at the cellular and network level, clinical and neurophysiological effects, and perspectives for future research.

## 2. Cerebellar tDCS in Hereditary Ataxias

### 2.1. General Background

Transcranial direct current stimulation is an increasingly applied non-invasive brain stimulation technique that involves the administration of weak electric currents in the order of magnitude of a few milliamperes through rectangular, square, or round rubber electrodes. Although the “active electrode” is typically affixed to the scalp overlying the intended cortical target area, maximum electric field strengths are not necessarily observed directly below this target electrode [27]. Cerebellar tDCS appears to be an exception, as computational modeling studies have consistently demonstrated the highest field strength underneath the target electrode, irrespective of its position, with only negligible spreading of the current to neighboring regions [27,28,29]. Depending on the particular task, function, or symptom that one aims to modulate, the electrode is placed over the midline (in order to reach the vermis and both paravermal regions) or specifically over the right or left cerebellar hemisphere [30]. In the case of cerebellar tDCS, the reference electrode is usually positioned over the forehead or cheek (i.e., the cephalic frontopolar and buccinator montages, respectively) or over the upper arm (i.e., the extracephalic deltoid montage).

### 2.2. Cellular Mechanisms and Network Effects of tDCS

#### 2.2.1. Insights from Supratentorial Brain Regions

The proposed mechanisms underlying tDCS-induced neuromodulation of the cerebellum are largely derived from studies on primary motor cortex (M1) tDCS in humans [31,32], which themselves were based on early animal experiments [33,34]. Surface-negative polarization, which is generally considered the equivalent of cathodal stimulation, has been shown to reduce the spontaneous firing rate of cortical neurons, while surface-positive polarization (i.e., generally considered the equivalent of anodal stimulation) increased it. Notably, the passage of currents for a brief period of only five to ten minutes caused prolonged after-effects on neuronal firing rate and evoked potential size, outlasting the duration of stimulation [33]. Similar polarity-dependent changes in cortical excitability were later confirmed in the human motor cortex by Nitsche and Paulus using tDCS and measurements of motor-evoked potential (MEP) amplitudes [31,32].

Empirical evidence indicates that subthreshold membrane potential shifts underlie the immediate effects of tDCS [35]. Although anodal stimulation is frequently referred to as “excitatory” and cathodal stimulation as “inhibitory”, this assumption seems to be an oversimplification of the biological reality. The actual direction of excitability shifts (i.e., increasing the likelihood of depolarization or hyperpolarization) depends on the spatial orientation of neurons relative to the applied electric field and on the specific neuronal compartment [34,36]. The ability of tDCS to elicit after-effects upon longer-lasting stimulation, on the other hand, has been related to changes in glutamatergic synaptic efficacy. In particular, administration of the *N*-methyl-D-aspartate (NMDA) receptor antagonist dextromethorphan abolished the prolonged effects of both anodal and cathodal tDCS, whereas the partial NMDA agonist D-cycloserine significantly enhanced the duration of MEP amplitude increases following anodal tDCS [37,38,39]. Further biochemical support for enduring alterations in the cortical excitation/inhibition balance in targeted brain regions is provided by MR spectroscopy studies [40,41,42]. Compared with baseline, a decrease in glutamatergic activity was observed after application of cathodal stimulation [42], while combined glutamine and glutamate levels increased following anodal stimulation [40]. In addition, anodal tDCS over M1 induced local reductions in gamma-aminobutyric acid (GABA) concentrations, which, interestingly, were found to correlate with the estimated average electric field strength [43,44] and motor learning performance [45,46]. The association with behavioral outcomes may suggest that local changes in the levels of this inhibitory neurotransmitter, generated by transcranial electrical stimulation, facilitate the occurrence of glutamatergic plasticity, at least in M1 [47]. Besides GABA, other neurotransmitters such as dopamine, acetylcholine, and norepinephrine are thought to play a modulatory role [48].

NMDA receptors, which are ligand-gated cation channels, have thus been proposed to importantly contribute to the after-effects of tDCS, and parallels were drawn with long-term potentiation (LTP) and long-term depression (LTD) in animal models [35]. Upon their activation by glutamate, the amplitude, duration, and location of the resulting calcium influx determine the direction of synaptic plasticity through different signaling cascades [49,50]. Whereas large and rapid elevations of intracellular calcium concentrations trigger early phase LTP, more moderate and slow increases will give rise to LTD [50,51]. Moreover, it has been demonstrated that influxes of intermediate magnitude (i.e., between the ranges required to produce either LTD or LTP) and very high intracellular calcium concentrations (i.e., above the range required to produce LTP) may not induce neuroplasticity at all or even cause changes in the opposite direction [51,52,53]. These zones are aptly referred to as “no man’s land” and may provide an explanation for the partial non-linearity of tDCS effects observed in studies that used relatively long-duration or high-intensity protocols [51,54,55,56,57].

Early tDCS investigations reported changes in the excitability of M1 lasting for approximately one hour after its application [32]. For clinical purposes, much more extended benefits are obviously needed, and it was hypothesized that these might be achieved through the administration of repeated sessions. Indeed, studies in patients with depression and stroke revealed that multiple tDCS sessions within a time period of five to ten days induced symptomatic improvements that were still present two to six weeks from baseline [58,59,60]. This concept of cumulative treatment exposure was based on the results from animal experiments showing (1) a sustained increase in neuronal firing rate when polarizing currents were delivered during the after-effects of a previous stimulation session and (2) the abolishment of such after-effects following local application of drugs that inhibit protein synthesis [61,62]. Monte-Silva and colleagues comprehensively assessed the impact of a second session of 1 mA anodal and cathodal tDCS over M1 and the possible influence of interstimulation interval in healthy individuals in terms of MEP amplitudes. Although the duration and patterns of excitability changes differed between both polarities, findings were generally in line with animal research, suggesting enhanced efficacy when a second session is administered during the after-effects of the first [53,63]. As potential explanations for the cumulative effects of anodal and cathodal stimulation, these authors drew parallels with late-phase LTP-like plasticity and LTD-like plasticity, respectively, hinting at possible alterations in postsynaptic receptor density and protein synthesis. However, similarly prolonged after-effects of a second session of cathodal tDCS compared with a single session could not be replicated by the same group, arguing against the induction of late-phase LTD by only one repetition [64]. Further work is required to identify the specific underlying physiological processes and to evaluate whether these differ across brain areas.

As a final remark in the discussion on the after-effects of tDCS (and other non-invasive brain stimulation techniques), the role of state-dependency as a source of interindividual variability should not be overlooked [65,66]. In other words, the occurrence of plastic changes is determined not only by properties of the external stimulus (e.g., current intensity, session duration, and repetition rate), but also by the state of neurons in the targeted brain region. Furthermore, subject-specific anatomical characteristics, such as sulcal depth, scalp-to-cortex distance, and skull thickness probably affect the magnitude and interindividual variation in after-effects, as these factors have been shown to explain a significant amount of the variability in electric field distribution [67,68]. It is important to note that the distinct cerebellar neuroanatomy—notably its dense, hierarchical folding and intricate cytoarchitecture—precludes a direct one-to-one translation of results from studies on M1 and other supratentorial regions to the cerebellum [69]. The mechanisms discussed in this section should therefore be regarded as possible explanations of how tDCS might affect the cerebellum.

#### 2.2.2. Evidence from Cerebellar tDCS

**Figure 1:**
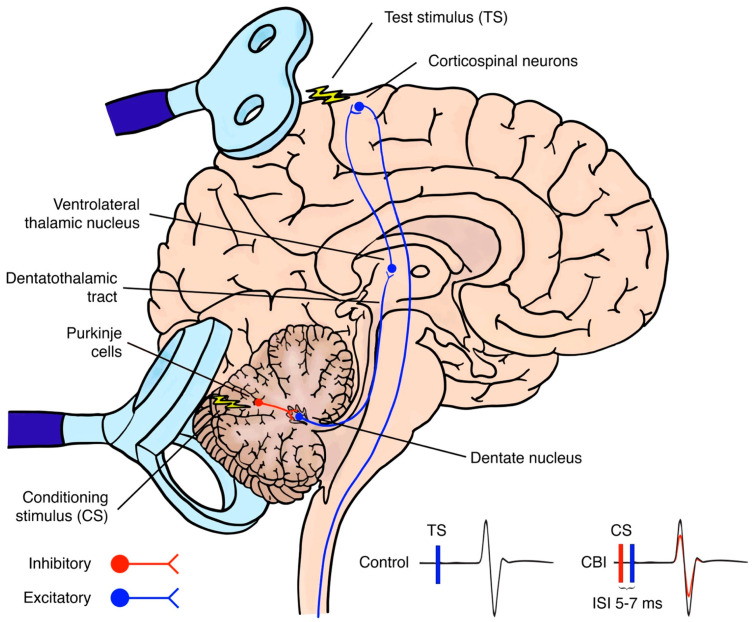
Midsagittal section illustrating the cerebellar brain inhibition paradigm. This paired-pulse transcranial magnetic stimulation protocol assesses the physiological inhibitory tone of the cerebellar cortex over the contralateral primary motor cortex. Specifically, the administration of a conditioning stimulus over one of the cerebellar hemispheres within a time interval of 5 to 7 ms prior to a test stimulus over the contralateral primary motor cortex leads to a decrease in motor-evoked potential amplitude.

At the individual neuronal level, early work in the turtle cerebellum has indicated that electrical stimulation alters the spike activity of Purkinje cell somata and dendrites depending on their orientation relative to the applied field. Current flow parallel to the somato–dendritic axis was found to depolarize or hyperpolarize the soma, while exerting the opposite effect on distal dendrites [70,71]. A recent computational modeling study suggested that the polarizing effects of cerebellar tDCS-induced electric fields are mainly limited to Purkinje cells [72]. The investigators reported that cerebellar tDCS (1) modulates the firing rate of these cells at rest in a polarity-specific manner through a change in somatic transmembrane voltage and (2) affects granule cells and neurons in the deep cerebellar nuclei to a much lesser extent. Further evidence that Purkinje cells probably constitute the primary target of cerebellar tDCS comes from TMS research using the cerebellar brain inhibition (CBI) paradigm. CBI is a paired-pulse TMS protocol that examines the functional connectivity of the cerebellothalamocortical pathway and indirectly reflects cerebellar excitability (Figure 1). When a conditioning stimulus (CS) over the (right) cerebellar hemisphere precedes a test stimulus (TS) over the contralateral M1 within an interval of 5 to 7 ms, the resulting excitation of Purkinje cells increases their inhibitory tone over the deep cerebellar nuclei, which subsequently leads to a diminished MEP amplitude [73,74,75]. Indeed, Galea and colleagues demonstrated that application of cerebellar cathodal tDCS decreased the ability of a cerebellar CS to elicit CBI, while anodal tDCS enhanced it, reinforcing the notion that this non-invasive stimulation technique changes Purkinje cell excitability in a polarity-dependent manner [76]. In accordance with these neurophysiological data, a recent fMRI study revealed increased activation of the dorsal dentate nuclei during and after cerebellar cathodal tDCS and a trend toward a reduction after anodal tDCS [77]. In summary, converging lines of research suggest that cerebellar tDCS modifies the inhibitory tone of Purkinje cells over the dentate nuclei, thereby influencing their output to the contralateral ventrolateral thalamus and M1.

A number of investigations in healthy adults found that tDCS over the cerebellum can also modulate cognitive and affective processing, presumably through a change in functional connectivity with cortical association areas and limbic regions. For instance, compared with anodal and sham stimulation, cathodal tDCS over the right cerebellar hemisphere enhanced performance of a mental arithmetic and verb generation task, which was attributed by the authors to disinhibition of the dorsolateral prefrontal cortex [78]. Furthermore, when applied over the midline, both tDCS polarities decreased reaction times for negative (but not positive and neutral) emotions in a facial expression recognition task, which provides support for a connection between the posterior vermis and limbic system [79]. Finally, anodal tDCS over the right cerebellum has been shown to increase functional connectivity with contralateral supratentorial regions implicated in reading and language processing [80]. These studies are just a few out of many examples to illustrate the potential of cerebellar tDCS to interfere with a variety of non-motor functions and also clearly demonstrate that the direction of outcomes is not predicted by stimulation polarity [81]. For a more comprehensive overview, the reader is referred to recent review articles on this particular topic [81,82].

### 2.3. Clinical and Neurophysiological Effects of Cerebellar tDCS

During the last ten years, an increasing number of studies have tested the hypothesis that modulating cerebellar cortical excitability with tDCS may induce clinical and neurophysiological changes in patients with degenerative ataxias.

#### 2.3.1. Single-Session Cerebellar tDCS

Grimaldi and Manto were the first to explore the effects of a single session of cerebellar anodal tDCS on short-latency and long-latency stretch reflexes, upper limb dexterity, and static posturography [83]. They included nine patients with hereditary and acquired types of ataxias and targeted the right cerebellar hemisphere or vermis with 1 mA for 20 min. Although no improvements in postural control and upper limb dexterity were found, amplitudes of long-latency stretch reflexes in the upper limbs decreased following anodal stimulation compared with sham stimulation. This was attributed by the authors to reduced disinhibition of the deep cerebellar nuclei resulting from increased Purkinje cell input. In order to establish patient-relevant changes in upper limb function, they speculated that stimulation should not be confined to the lateral cerebellum, but must more substantially modify cerebello-cerebral connectivity. In a second study, they therefore administered anodal tDCS (1 mA) to the right cerebellar hemisphere for 20 min, immediately followed by 20 min of anodal tDCS over the contralateral primary motor cortex (M1) in two individuals with SCA2 [84]. Using triaxial accelerometers and haptic technology, a significant reduction in postural tremor and action tremor amplitude was observed. In addition, fast goal-directed wrist movements were less hypermetric, as quantified by a decrease in the onset latency of the antagonist muscle activity. Although limited by the very small size, these findings seem to suggest that sequential cerebellum-M1 tDCS is able to transiently modulate oscillatory activity along a compromised cerebellothalamocortical pathway. A reduction in right arm postural tremor amplitude was also attained in a patient with autosomal recessive ataxia due to homozygous *ANO10* mutations when the anode was placed over the right cerebellar hemisphere and the cathode was simultaneously positioned over contralateral M1 [85]. Hypermetria also slightly decreased, but to a lesser degree than in the SCA2 patients.

Two randomized, double-blind, sham-controlled trials have examined the effects of midline cerebellar anodal tDCS (20 min, 2 mA, cathode over the right deltoid muscle, offline design) with an array of clinical outcome measures, including the Scale for the Assessment and Rating of Ataxia (SARA), International Cooperative Ataxia Rating Scale (ICARS), 8 m walk test (8MWT), and 9-hole peg test (9HPT) [86,87]. The first used a cross-over design and included nineteen individuals with SCA2, SCA1, SCA38, FRDA, AOA2, FXTAS, multiple system atrophy of cerebellar type (MSA-C), and sporadic adult-onset ataxia with unknown etiology (SAOA) [86]. Despite the heterogeneity of diseases, a significant treatment effect was found for all endpoints in favor of real tDCS. Overall, the investigators described a mean between-group difference of 1.40 points in SARA score, 4.37 points in ICARS score, 1.37 s in the 9HPT, and 1.42 s in the 8MWT, with similar results among SCA and MSA-C patients. The second trial included an etiologically homogeneous group of twenty SCA3 patients and investigated whether daily sessions of cerebellar tDCS for two weeks can induce long-term clinical improvement through cumulative effects (see below) [87]. As one of the secondary endpoints, SARA score, 9HPT, 8MWT, PATA repetition rate, and static posturography were also determined directly after the first session of tDCS. In contrast to the other study, this trial found no evidence of a transient symptomatic benefit of single-session tDCS.

Finally, based on previous work in healthy volunteers reporting an acceleration of motor learning with cerebellar anodal tDCS [88,89,90], German researchers examined whether similarly favorable outcomes of online stimulation could be obtained in patients with cerebellar disorders [91,92]. In their first study, which involved a force field reaching task, they did not show faster motor adaptation compared with sham tDCS or M1 tDCS in nineteen patients with hereditary and acquired ataxias [91]. In the second study, grip force control deficits remained unchanged following 2 mA to the right cerebellar hemisphere in a subset of fourteen patients [92].

#### 2.3.2. Repeated Sessions of Cerebellar tDCS

While the after-effects of single-session cerebellar tDCS, if any, are transient, lasting at most a few hours, repeated administration within a short time period has been hypothesized to generate beneficial results for days, weeks, or even months through cumulative changes in functional connectivity with M1 and cortical association areas (as previously shown in stroke and depression, see Section 2.2). Over the past five years, several randomized, double-blind, sham-controlled trials have explored the long-term therapeutic potential of a two-week protocol comprising daily sessions of midline cerebellar anodal tDCS or cerebello-spinal tDCS in degenerative ataxias.

Benussi and colleagues first evaluated the effects of cerebellar anodal tDCS (2 mA, 20 min) in twenty patients with SCA2, SCA38, SCA14, FRDA, AOA2, MSA-C, FXTAS, and SAOA [93]. They observed significant improvements in SARA score and ICARS score that persisted throughout the follow-up period of three months. The decrease in ataxia severity (1) specifically involved the ICARS domains of posture and gait and limb coordination, (2) was most pronounced in the least severely affected individuals, and (3) was associated with a significant increase in CBI that similarly persisted for three months. In apparent contradiction with these clinician-based outcome measures, the investigators did not find a treatment effect for quality of life. In a second trial with cross-over design, they tested the effectiveness of cerebello-spinal tDCS (2 mA, 20 min, cathode 2 cm below the eleventh thoracic vertebra) in twenty patients with the same etiologies as in the first study [94]. Results were comparable, including the correlation between clinical improvement and change in CBI, inverse association with ataxia severity at baseline, and duration of effects. There were no differences between individuals with MSA-C and those with SCA. In parallel with a mean reduction in SARA score of more than four points, the investigators now also reported a significant increase in quality of life. Recently, they published the results of a second trial of cerebello-spinal tDCS (2 mA, 20 min), which contained both a randomized, double-blind, sham-controlled phase and an open-label extension phase after three months, in which all 61 participants received real tDCS daily for two weeks [95]. The causes of ataxia were mixed again, including SCA1 (*n* = 5), SCA2 (*n* = 12), SCA14 (*n* = 1), SCA28 (*n* = 1), SCA38 (*n* = 5), FRDA (*n* = 7), CANVAS (*n* = 3), MSA-C (*n* = 10), and SAOA (*n* = 17). Regardless of the underlying disease category, improvements were found in ataxia severity, cerebellar cognitive affective syndrome scale (CCAS-S) score, quality of life, and CBI, with an add-on effect of a second treatment round of cerebello-spinal tDCS after three months. In line with their previous work, (1) there were significant associations between clinical and neurophysiological changes and (2) benefits were greatest in individuals with the least severe ataxia.

Promising results of cerebellar tDCS in mixed ataxia cohorts could not be corroborated in a recent SCA3 trial [87]. Driven by the consistent inverse correlation between baseline disease severity and delta SARA score, Maas and colleagues specifically included mildly to moderately affected patients. From a methodological perspective, electrode size, montage, current intensity, and session duration were identical to those applied previously [93]. Nonetheless, there were no short-term or long-term differences between groups in SARA score, CCAS-S score, and CBI. In addition, there were no significant treatment effects for various static posturography parameters, walking speed, manual dexterity, activities of daily living, health-related quality of life, depressive symptoms, physical activity, and direct medical costs. Differences in SARA speech subscore after six months and the number of extracerebellar signs after three and six months were the only endpoints that reached statistical significance in favor of real tDCS. Interestingly, however, some of the participants in the intervention arm showed a relevant reduction in SARA score that lasted six or even twelve months, which likely indicates interindividual variability in treatment response. Although none of the baseline parameters correlated with change in SARA score, associations may have been masked by the relatively small sample size, and further studies are needed to detect individual predictors of improvement. Discrepancies in the overall conclusions between this trial and the previous ones may be due to significant differences in the disease severity of their participants, the well-established interindividual variation in tDCS-induced effects, and/or disease-related factors (e.g., prominent degeneration of the dentate nuclei in SCA3, which may not be overcome by cerebellar cortical stimulation). Furthermore, the observation of significant short-term improvements in various outcome measures in sham-treated individuals was striking and might reflect a placebo effect resulting from inappropriately high expectations of benefit [96]. Notably, besides clinical endpoints, possible changes in motor learning were examined using a delay eyeblink conditioning paradigm that relies on the functional integrity of the cerebellar cortex and interposed nuclei [97]. Although multiple sessions of cerebellar anodal tDCS did not significantly enhance the acquisition of conditioned eyeblink responses (CRs), a treatment effect was found for timing. Onset and peak latencies of CRs were significantly longer in the real tDCS group compared with baseline, indicating that this intervention can modulate cerebellar temporal processing.

Finally, two case reports with an open-label design have suggested favorable results of cerebellar tDCS in patients with degenerative ataxia [98,99]. The first involved a woman with progressive ataxia of unknown etiology who received 60 sessions of remotely supervised tDCS over the midline cerebellum (2.5 mA, 20 min) paired with cognitive training and followed by physical therapy [98]. Improvements in gait speed, manual dexterity, and self-reported fatigue were described. Although general learning effects of repeated assessment may have played a role, especially in the pegboard test, and the study was not an n-of-1 trial, it importantly demonstrates the feasibility of extended self-administration of tDCS at home under strict clinical supervision. In the second case report, online administration of anodal and cathodal tDCS (2 mA, 10 min) during 24 sessions of robotic gait rehabilitation both led to a reduction in SARA score and an increase in CBI in a patient with FRDA, while no relevant changes were found after robotic training only [99].

### 2.4. Future Perspectives

The tDCS studies discussed in the previous section were highly heterogeneous regarding (1) the etiology of cerebellar ataxia (i.e., rather pure cerebellar cortical degeneration versus combined involvement of the cerebellar cortex and deep cerebellar nuclei), (2) disease severity in terms of SARA score and performance measures, such as the time required to complete the 8MWT and 9HPT, (3) specific aim and selected primary endpoint (i.e., clinically oriented versus focused on more fundamental aspects), (4) the applied current intensity (i.e., 1, 2, or 2.5 mA), (5) session duration (i.e., mostly 20 min, but ranging from 10 to 25 min), (6) repetition rate, (7) position of the target electrode (i.e., covering the midline or specifically targeting the right cerebellar hemisphere), (8) position of the reference electrode (i.e., frontopolar, buccinator, deltoid, and spinal montages), (9) size of the electrodes (i.e., 5 × 7 cm, 5 × 5 cm, and 5 × 4 cm), and (10) design (i.e., a between-subjects study in which participants received real tDCS *or* sham tDCS versus a within-subjects study in which they received both types of stimulation after a wash-out period). All these differences impede the comparability and interpretation of results, leaving many questions unanswered. For which types of cerebellar ataxia and for which disease stage may repeated sessions of cerebellar tDCS lead to symptomatic improvement? To what extent does interindividual variability in treatment response play a role in patients with ataxia and which factors might predict favorable effects? How much of the current actually reaches different cerebellar regions in patients with various degrees of atrophy? What is the influence of the position of the reference electrode? Which treatment schedules are optimal in terms of session duration, number of sessions, and repetition rate? Should individualized dosing schemes be used? Is cerebellar anodal tDCS better than cerebello-spinal tDCS?

With the exception of one study, all randomized, double-blind, sham-controlled trials conducted thus far involved individuals with mixed etiologies. Despite the rarity of these disorders, future research would greatly benefit from including (larger numbers of) patients with the same type of ataxia.

Modeling studies that estimate the strength and spatial distribution of the electric field induced by cerebellar tDCS are currently lacking for patients with ataxias, but may provide important novel insights and answers to some of the questions listed above. Converging evidence indicates that motor, cognitive, and affective functions are subserved by distinct cerebellar regions that project to various supratentorial areas, yielding specific cerebello-cerebral loops that might be differentially amenable to the effects of cerebellar stimulation [100]. For instance, motor representations of the face, arm, and leg are located in the anterior lobe, part of lobule VI, and lobule VIII, which appear more difficult to reach from outside due to their inward folding. From an anatomical perspective, Crus I and II seem to be the major targets of cerebellar tDCS, leading to the suggestion that the frequently disturbed cognitive and emotional processing in patients with cerebellar disorders may be more easily and effectively modulated than motor deficits. Further trials with a primary focus on cognitive and affective functions are required to examine this anatomy-driven hypothesis.

Finally, although there are numerous challenges for conducting trials in patients with degenerative ataxias, the implementation of remotely supervised home-based tDCS is an exciting development for those with impaired mobility and/or cognition [101]. It will allow the administration of cerebellar tDCS for extended periods of time without the burden of travel to specialized centers, improving the feasibility of clinical trials in this burgeoning area of research.

## 3. Cerebellar TMS in Hereditary Ataxias

### 3.1. General Background

Transcranial magnetic stimulation is a non-invasive brain stimulation method that employs the principle of electromagnetic induction to generate currents in specific areas of the brain, allowing for targeted stimulation of neuronal populations [102]. It is applied both in research and clinical settings and has been proven effective in treating a variety of conditions, including depression, chronic neuropathic pain, stroke, and several other neurological and psychiatric disorders (i.e., either possible, probable, or definite efficacy according to recently updated consensus recommendations) [103]. The main difference between TMS and tDCS is the type of energy used to stimulate the brain. TMS makes use of magnetic fields to induce an electric current in the brain, causing depolarization of neuronal populations and eliciting action potentials, while tDCS (as discussed previously) makes use of a direct electric current that increases or decreases the likelihood of spontaneous neuronal firing [104]. TMS is generally more focal and capable of targeting deeper brain structures more effectively, while tDCS is more diffuse and less able to target specific regions. TMS is also typically more expensive and less portable compared with tDCS [104].

### 3.2. Cellular Mechanisms and Network Effects of TMS

Repetitive TMS (rTMS) aims to modulate cortical activity beyond the period of stimulation, making it a potential treatment option [103,105]. It can be applied at various frequencies or as a patterned train of pulses over motor and non-motor brain regions [102]. In general, stimulation at frequencies lower than 1 Hz leads to decreased activity in the targeted area, while frequencies above 5 Hz tend to increase activity [105,106]. However, there is considerable interindividual variability in the response to rTMS. Another popular rTMS variant, theta burst stimulation (TBS), delivers pulses more efficiently in bursts consisting of 50 Hz pulses at 5 Hz. Continuous theta burst stimulation (cTBS) is thought to inhibit neural excitability, whereas intermittent theta burst stimulation (iTBS) is thought to enhance it. Similar to tDCS, after-effects are attributed to synaptic plasticity, which is likely achieved through processes similar to LTP or LTD [107,108]. Such changes are believed to restructure the neural network of patients with neurological diseases, facilitating their rehabilitation process [109].

Besides its potential therapeutic applications, TMS has been widely used to examine corticospinal tract and motor cortex dysfunction in hereditary cerebellar ataxias and may provide neurophysiological biomarkers to track disease progression over time [110]. One such paradigm, the previously introduced CBI protocol, appears particularly promising as a (monitoring) biomarker, as (changes in) the degree of MEP amplitude inhibition following a cerebellar CS were found to correlate with (changes in) ataxia severity [73,93,94,95,111,112,113,114,115,116,117].

The cerebellum is traditionally thought of as being primarily involved in motor control and coordination. However, more recent research has revealed that it is also intimately connected with a variety of supratentorial non-motor areas, including the prefrontal cortex, parietal cortex, and temporal cortex [118]. These connections imply that the cerebellum plays a modulating role in a number of cognitive and emotional processes, such as executive functions, language, and social cognition [119]. Interestingly, several studies in healthy individuals have shown that cerebellar rTMS may improve attention, working memory, and decision making, presumably by inducing changes in the connectivity with supratentorial regions. It has also been shown to modulate emotional processing, as evidenced by a reduction in anxiety and enhancement of mood. Furthermore, cerebellar rTMS may influence social cognition, especially the processing of social cues [24,120].

In recent years, various new methods for cerebellar TMS have been developed, utilizing different coil shapes and stimulation protocols. These methods have led to a range of stimulation effects that require further investigation. The most successful type of coil and recommended treatment regimens for achieving measurable neuroplastic changes and behavioral improvement should be evaluated systematically through validated and clinically meaningful rating scales. However, there is a general lack of high-quality randomized controlled trials examining the effects of cerebellar TMS [121].

### 3.3. Clinical and Neurophysiological Effects of Cerebellar TMS

Shimizu and colleagues were the first to study the effects of low-frequency rTMS in four patients with different types of SCA (i.e., two with SCA6, one with SCA1, and one with SCA7). These investigators applied ten TMS pulses at 100% of maximum stimulator output and an interpulse interval of more than five seconds over both cerebellar hemispheres and the inion for twenty-one consecutive days. Compared with baseline, they observed an increase in walking speed, number of steps in tandem gait, and blood flow in both cerebellar hemispheres, putamen, and pons, as determined with SPECT [122]. In a subsequent double-blind, sham-controlled trial by the same group, 74 patients with sporadic or hereditary “spinocerebellar degeneration” underwent a similar protocol as in their previous study. Although there were clear placebo and/or training effects, the intervention group displayed a greater improvement in gait speed and standing capacities than the sham group, as well as an increase in mean regional blood flow in the cerebellum and pons. Notably, when rTMS was continued once or twice a week after completing the trial, the improvement was found to persist for at least 6 months. On the other hand, patients receiving rTMS once every 2 weeks quickly returned to their baseline condition [123]. Finally, Ihara and colleagues applied the same treatment regimen for eight weeks in twenty individuals with spinocerebellar degeneration (i.e., ten with “olivopontocerebellar atrophy”, six with “cortical cerebellar atrophy”, and four with SCA6). Oxidative stress biomarkers decreased in most patients, which correlated with a reduction in ICARS scores. Although cerebrospinal fluid concentrations of ascorbate free radical were significantly higher at baseline in patients compared with a group of healthy subjects, they declined following rTMS and were no longer different from these controls [124]. Oxidative stress is thought to play a role in the pathology of several types of ataxia [125,126], with in vitro studies showing a neuroprotective effect of rTMS [127].

Manor and colleagues recently conducted a randomized, double-blind, sham-controlled trial in twenty patients with genetically confirmed SCAs in which they applied twenty sessions of neuronavigation-guided cerebellar rTMS over four consecutive weeks. Ten pulses were administered over each hemisphere and the vermis, making a total of 30 pulses per session. Compared with sham stimulation, the investigators found that rTMS resulted in a larger decrease in SARA score after one month, but not after one week, particularly within the stance item. This improvement was paralleled by reductions in postural sway speed and area. However, rTMS did not affect performance of the 9HPT, timed up-and-go test, or gait kinematics [128].

In another randomized, double-blind, sham-controlled, cross-over trial that included 24 patients with SCA3, MSA-C, and post-lesion ataxia, França and colleagues administered 1 Hz rTMS over the cerebellar hemisphere contralateral to the most affected side. The protocol comprised 1200 pulses per session, which were delivered by a double-cone coil during five consecutive days. The authors observed a significantly larger reduction in SARA and ICARS scores compared with sham stimulation, particularly in the “kinetic function” subscore, but only in individuals with MSA-C. There was no significant carry-over effect in SARA and ICARS scores, indicating that the possible benefits of the active sessions did not persist after the wash-out period of four weeks [129].

The effects of cerebellar rTMS in individuals with SCA3 were further investigated in two studies from the same group. Chen and colleagues conducted a randomized, double-blind, sham-controlled trial in eighteen patients, applying 30 min of 1 Hz rTMS (for a total of 900 pulses) over the inion during fifteen consecutive days. They observed a significant decrease in ICARS scores in both groups, which was more prominent in the intervention arm. Moreover, values of N-acetyl aspartate (NAA)/creatine (Cr) and choline (Cho)/Cr in the cerebellar vermis, dentate nuclei, and cerebellar hemispheres, as evaluated with MRS, increased significantly only after real stimulation. Finally, a negative correlation was found between the change in NAA/Cr in the right cerebellar hemisphere and the change in ICARS scores [130]. A subsequent larger randomized, double-blind, sham-controlled trial was carried out in 44 patients with SCA3, using a similar protocol as in the previous study but with a different target (i.e., both cerebellar hemispheres, making a total of 1800 pulses). After fifteen days of treatment, the investigators reported significant changes in SARA score, ICARS score, and Berg Balance score in both groups, again pointing to an important placebo or training effect, but improvements were larger in individuals assigned to real stimulation [131].

Another randomized, double-blind, sham-controlled, cross-over trial evaluated the effects of ten sessions of iTBS (with the coil positioned 1 cm below and 3 cm left/right to the inion) in six patients with SCA38. After ten sessions of real cerebellar iTBS, modified ICARS scores decreased. At the group level, there was no noticeable change in serum brain-derived neurotrophic factor (BDNF) concentration. However, upon segregation of samples by genotype, BDNF levels were found to have increased in all three individuals with the Val66Val genotype and decreased in the three patients with the Val66Met genotype [132].

Two case reports with an open-label design have suggested favorable results of repeated sessions of cerebellar TMS in SCA6. Dang and colleagues administered twenty sessions comprising 1500 pulses over the inion in 1 s trains at a frequency of 10 Hz with a 10 s intertrain interval. SARA score decreased from 14 points at baseline to 6 points after the intervention and to 2.5 points after eighteen months (without additional therapy). Improvements were observed in the ICARS domains of posture and gait, limb coordination, and speech, but not in oculomotor movements [133]. In the second case report, TMS was applied over both M1 and the cerebellum five days a week for two consecutive weeks, with a repetition of the protocol after two weeks. Specifically, the investigators administered 40 single pulses at approximately 0.3 Hz over M1 (i.e., twenty with the current flowing counter-clockwise and twenty with the current flowing clockwise, holding the circular coil over Cz), followed by twenty single pulses at 0.5 Hz over the inion (i.e., ten with the current flowing counter-clockwise and ten with the current flowing clockwise). They described a significant improvement in diplopia, particularly after M1 stimulation, along with an improvement in limb ataxia, as evaluated using the ICARS [134].

On a different note, Lin and colleagues studied the impact of a single session of cTBS over the right cerebellar hemisphere on the auditory–vocal integration of nineteen patients with SCA1, SCA2, SCA3, and SCA6. Compared with sham stimulation, they found smaller vocal adjustments for pitch changes, increased cortical P1 and P2 responses, and decreased N1 responses following real cTBS. Furthermore, individuals with larger amplitudes of P1 and P2 responses had smaller adjustments in their vocal pitch. These results imply that cerebellar cTBS can modulate abnormal auditory–motor integration for vocal pitch regulation in patients with SCA [135].

Finally, a recent meta-analysis including seven randomized controlled trials concluded (1) that cerebellar rTMS significantly improved SARA, ICARS, and Berg Balance scores, (2) that high-frequency stimulation was most effective, (3) that real and sham stimulation did not differ in the incidence of adverse effects, and (4) importantly, that overall evidence is limited [136].

### 3.4. Future Perspectives

Similar to tDCS (see also Section 2.4), studies on cerebellar rTMS were highly heterogeneous in terms of ataxia etiology and stimulation protocol (e.g., type of coil, frequency, session duration, number of sessions, position of the coil, and outcome measures), making it difficult to compare their results. In order to improve the quality of data, future research needs to take these factors into account in a systematic manner. Because of the etiological heterogeneity and small sample size of most trials, it remains unclear which patients might benefit from the application of cerebellar rTMS. Further studies are required in larger numbers of individuals with the same type of ataxia.

A second objective for future research on cerebellar rTMS should be to obtain a more comprehensive understanding of the underlying mechanisms that contribute to its therapeutic effects in patients with ataxia. This includes investigating the specific neural pathways and cellular processes that are affected by rTMS, as well as how such changes may actually result in clinical improvements in motor, cognitive, and/or affective functions. Importantly, more targeted and effective treatment protocols need to be developed by carefully selecting the optimal parameters, such as site, frequency, and duration of stimulation.

Another aspect that requires further investigation is the long-term effectiveness (and safety) of different rTMS regimens. As most studies thus far focused on short-term outcomes, additional research is warranted to examine the durability of the therapeutic effects of rTMS over time and how protocols might be adjusted to maintain symptomatic improvement. Moreover, the development of miniaturized devices, which have already been tested in patients with depression [137], may allow the application of cerebellar rTMS in a home setting, which could greatly increase its accessibility and facilitate clinical trials.

## 4. Cerebellar DBS in Hereditary Ataxias

### 4.1. General Background

Deep brain stimulation is an invasive form of neuromodulation in which a quadripolar or octopolar electrode is implanted in a specific brain region. It has proven to be an effective treatment strategy for a wide range of neurological and psychiatric disorders, such as Parkinson’s disease, essential tremor, dystonia, Tourette syndrome, obsessive compulsive disorder, neuropathic pain, cluster headache, and epilepsy [138,139,140,141,142,143]. Furthermore, DBS is currently under investigation for numerous other conditions, including Alzheimer’s disease, Huntington’s disease, traumatic brain injury, other pain syndromes, bipolar disorder, schizophrenia, depression, and addiction [144].

Clinical effects of DBS are intimately related to the area in which the electrodes are positioned. The basic principle involves modulation of a brain region in which multiple fibers implicated in the pathogenesis of the particular disease are located. For each disorder, there is an optimal hub or hotspot, which needs to be targeted in order to obtain symptomatic improvement [144]. However, even in a single disease, one may select different targets depending on the patient’s predominant symptom. For example, individuals with Parkinson’s disease who require large amounts of dopaminergic medication and experience motor fluctuations might benefit most from DBS in the subthalamic nucleus (STN), while globus pallidus internus (GPI) DBS could be more suitable for patients with troublesome dyskinesias at lower doses [145].

### 4.2. Cellular Mechanisms and Network Effects of DBS

Despite its widespread use, the mechanisms of action of DBS at the cellular and network level remain incompletely understood. Favorable results were initially hypothesized to stem from a functional lesion, reducing the overactivity of neuronal populations in the chosen target area. Although this theory may provide an explanation for why motor outcomes following GPI and thalamic DBS are generally similar to those of pallidotomies and thalamotomies, it cannot account for all clinical and neurophysiological observations and is therefore regarded as an overly simplistic view [146]. It has been demonstrated, for instance, that high-frequency GPI and STN DBS, which improve symptoms in Parkinson’s disease, increase GPI output [147,148], but there is also evidence that GPI overactivity is one of the causes of symptoms in this disease [149,150].

Effects of DBS on downstream structures in terms of neuronal inhibition or excitation can be quite opposite to those in the stimulated region directly surrounding the electrode tip [147,148,151]. Indeed, electric currents may hyperpolarize cell bodies and dendrites while generating action potentials at the axonal level, causing local inhibition and outflow excitation at the same time [152]. Rather than just inhibition or just excitation, both processes most likely occur simultaneously in different neuronal structures [153]. In addition to local and immediate downstream effects, DBS has been shown to modulate the activity patterns of neural networks [146]. These network effects may explain why certain symptoms, such as dystonia, take longer to improve after DBS and, similarly, take longer to worsen when stimulation is stopped [154].

Over the last number of years, much attention has been directed to DBS disrupting pathological frequency patterns. The most comprehensively studied example concerns the exaggerated beta activity (13–30 Hz) in the STN of patients with Parkinson’s disease, which is linked to rigidity and bradykinesia. Clinical improvement of these symptoms after DBS was paralleled by a reduction in the spectral power of this beta band [155]. Other examples include an excess of beta activity in the STN in individuals with focal dystonia and an average low-frequency oscillatory power (4–12 Hz) in the GPI, correlating with symptom severity across many types of dystonia [156]. The correction of such aberrant frequency patterns might help explain the efficacy of DBS in many neurological and psychiatric diseases.

### 4.3. Clinical and Neurophysiological Effects of Cerebellar DBS

Prior to its experimental application in subjects with ataxia, cerebellar DBS has been investigated in animal models and patients with stroke. One of the first studies in this field showed that 20 Hz stimulation of the dentate nucleus improved motor function in rodents after a stroke affecting the contralateral sensorimotor cortex [157]. Subsequent work by the same group revealed that dentate nucleus DBS, especially at a frequency of 30 Hz, was able to modulate motor cortex excitability, enhance perilesional expression of synaptophysin, and increase neurogenesis in the perilesional motor cortex [158,159,160]. Therapeutic efficacy of cerebellar DBS has also been explored in a handful of patients after cerebellar stroke, with some evidence of clinical improvement [161,162,163]. Results from clinical trials in patients with middle cerebral artery stroke are still pending (e.g., NCT02835443).

Effects of cerebellar DBS have been examined in various animal models of degenerative ataxia using different targets [164,165,166]. Many types of degenerative ataxia are characterized by loss of Purkinje cells, which leads to an altered output to the deep cerebellar nuclei [167]. Accordingly, modulation of the latter nuclei through electrical stimulation has been hypothesized to change firing patterns and induce symptomatic improvement. However, because of the very small size of the dentate and interposed nuclei in rodents and the proximity of other structures, it is hard to prove definitively that beneficial effects result solely from stimulation of the targeted nucleus and not partly from the spreading of currents [165].

Anderson and colleagues implanted electrodes in the dorsal dentate nuclei of seven *shaker* rats (i.e., an X-linked recessive rodent model of degenerative cerebellar ataxia) and compared ON vs. OFF-stimulation conditions regarding tremor, straightness of gait, and fall rates [165]. Pulse width was set at 100 µs, current intensity was chosen based on the threshold to induce side effects (90%), and frequencies ranged from 4 to 180 Hz. The investigators observed a significant reduction in tremor using 10, 20, and 30 Hz stimulation on an acute timescale of five minutes and using 30 Hz stimulation on a moderate timescale of two hours. Furthermore, straightness of gait was found to improve following 20 and 30 Hz stimulation and to worsen following 130 and 180 Hz stimulation. Only 30 Hz stimulation decreased fall rates, but this result was statistically significant only with short-duration stimulation.

Miterko and colleagues examined DBS of the interposed nuclei in a specific mouse model of hereditary ataxia, in which cells do not degenerate but cerebellar circuit function is altered [164]. They reported an increase in the latency to fall on an accelerating rotarod in twelve ataxic mice after 13 Hz, but not after 2 Hz or 130 Hz stimulation. Moreover, only beta-frequency DBS improved muscle function during walking, field exploration during the open field assay, and stepping. Interestingly, step cycle changes were sustained seven days after DBS was stopped.

**Figure 2:**
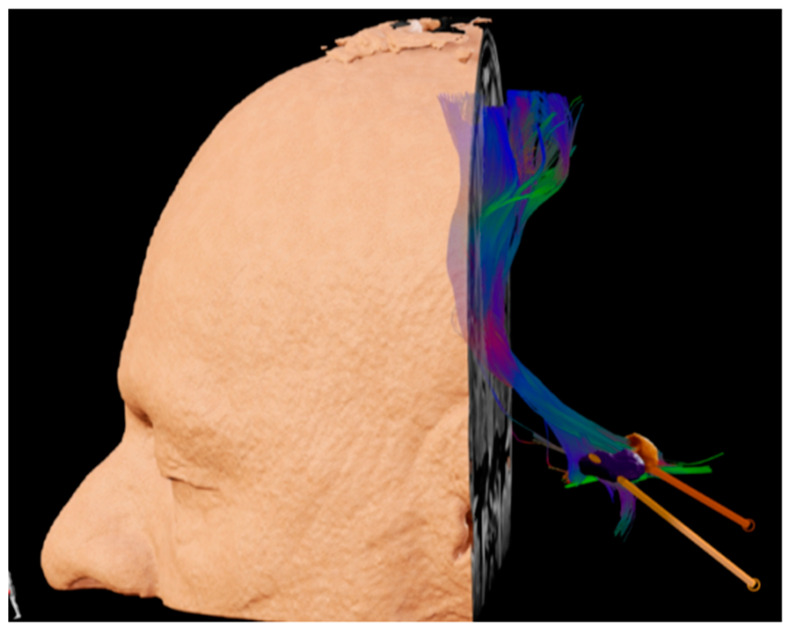
Post-operative image control of dentate nucleus DBS. Sagittal T1-weighted MRI reconstruction of DBS electrodes in the left (purple volume) and right (orange volume) dentate nucleus, with a close relation to the ascending dentato-rubro-thalamic tract. Reconstructions and tractography were performed with Elements Software (Brainlab AG, Munich, Germany).

To our knowledge, there is only one small human ataxia cohort in which the safety and efficacy of cerebellar DBS have been explored [163,168,169]. Cury and colleagues conducted a randomized, double-blind, crossover pilot study using bilateral dentate nucleus DBS in two patients with SCA3, two patients with a cerebellar stroke, and one patient with ataxia and tremor due to cerebral palsy [163]. A total of fourteen subjects had received 1 Hz cerebellar rTMS beforehand, and only good responders (i.e., defined by an improvement in SARA score of 30% or more) were deemed eligible for DBS surgery. Patients were operated in prone position under general anesthesia. The tip of the electrode was aimed at the ascending dentato-rubro-thalamic tract, while the remaining three contacts were in the anterior and superior parts of the dentate nuclei (Figure 2) [168]. No serious adverse events occurred. Four out of five patients showed an improvement in ataxia and tremor severity after active stimulation, as quantified by the SARA and Fahn-Tolosa-Marin Tremor Rating Scale (FTMTRS). The mean difference in change in SARA score from baseline between active and sham stimulation was 1.5 points, which did not reach statistical significance. Patient global impression of change and decreases in FTMTRS score were significantly different between both types of stimulation. DBS parameters varied widely. In individuals with SCA3, lower frequencies of 16 Hz and 8 Hz were used in a bipolar configuration, while the two patients with cerebellar stroke improved after high-frequency monopolar stimulation with 104 Hz.

Stimulation frequency is a pivotal aspect of DBS in various movement disorders. In Parkinson’s disease, STN DBS is typically applied with high frequencies (i.e., >100 Hz), but lower frequencies above 60 Hz have already been shown to effectively reduce bradykinesia and decrease stimulation-induced side effects regarding gait and speech [170,171]. In addition, symptom control in essential tremor is usually better when higher frequencies are administered [172]. In general, low-frequency electrical stimulation enhances network flow in neural tissues [157,173], while high-frequency stimulation mimics a lesion effect and impairs neuronal communication [174,175,176]. Because the objective of DBS in Parkinson’s disease and essential tremor is to override pathological oscillatory activity, it makes sense to use higher frequencies in both disorders [177]. However, in degenerative ataxia models characterized by loss of Purkinje cells, this might be different. Considering (1) that these neurons enable the execution of well-timed, accurate movements through connections with deep cerebellar nuclei, (2) that interruption of their activity impairs such movements, and (3) that decreases in their numbers generate a chronic disinhibition of the deep nuclei, impairing communication flow, one may envisage a role for low-frequency DBS to improve, rather than undermine, cerebellar network throughput in patients with cerebellar cortical degeneration. Interestingly, out of a range of frequencies from 4 to 180 Hz, 30 Hz was found to be optimal in improving ataxic symptoms in a rat model of Purkinje cell loss, while a worsening of incoordination was observed with frequencies above 100 Hz [165].

### 4.4. Future Perspectives

It follows from the preceding section that the field of cerebellar DBS is still in its infancy. Stimulation targets and protocols were quite different between animal studies, and there has been only one small cohort of patients with ataxia exposed to this intervention. Further research on its safety and efficacy is obviously required in larger numbers of ataxia patients, preferably with the same etiology, in order to draw more robust conclusions.

The definition of the right target region is key to the success of DBS. Because of the heterogeneity of degenerative cerebellar ataxias, a potentially suitable structure for one specific disorder might not necessarily be the best target for another. In this regard, the use of a single electrode reaching more than one structure, depending on the trajectory, could be helpful in determining the hotspot [178]. Studies in humans thus far only involved dentate nucleus DBS, while animal research also explored (and demonstrated favorable outcomes following) stimulation of the interposed nuclei.

Part of the variability in the observed clinical effects may be explained by differences in ataxia severity at the time of treatment. Indeed, animal studies have demonstrated that mice with less severe ataxia tended to improve after cerebellar DBS, while the more severely affected mice did not benefit [164]. Future investigations should look for predictive factors that might differentiate responders from non-responders.

A clear advantage of DBS is the possibility to preset groups of parameters, which allows patients to shift between programs at home. This could facilitate a clinical comparison between different electrophysiological parameters and, depending on the DBS target, a comparison of hotspots.

Notably, it is currently possible to reconstruct the location of the lead inside a patient’s brain with trustworthy and readily accessible software [179,180,181]. Such tools enable a detailed visualization of the induced electric fields around the lead and enhance our understanding of which structures are actually being stimulated.

Finally, as discussed above, the optimal frequency of stimulation in different types of ataxia is a matter of debate. Following the example of Parkinson’s disease and dystonia, an exciting future perspective in ataxias would be the application of devices capable of reading local field potential signals [155,156]. This development may not only aid in DBS programming, but would also help us better understand the pathophysiology of ataxic disorders.

## 5. Conclusions

Effective symptomatic and/or disease-modifying therapies are currently lacking for the majority of degenerative cerebellar ataxias. Over the past decade, several brain stimulation techniques, including rTMS and tDCS and, to a lesser extent, dentate nucleus DBS, have shown promise in modulating cerebellar excitability and restoring physiological activity in patients with ataxia and other neurodegenerative disorders. However, a significant limitation of the studies conducted thus far that precludes robust inferences is the considerable degree of variability in their methodology. As discussed, there were large differences in the number of sessions, site of stimulation, current intensity or coil type, and outcome measures. Furthermore, the rarity of most cerebellar ataxias has restricted the ability to perform large-scale clinical trials in etiologically homogeneous groups of patients. Finally, publication bias, the tendency to preferentially publish positive or statistically significant results, is a salient issue, with neutral findings probably not being reported as often by investigators and scientific journals. We would like to emphasize the importance of well-conducted trials with neutral results to move the brain stimulation field forward and encourage both researchers and journals to publish outcomes of decent work regardless of the direction of effect.

Although various studies have suggested that non-invasive cerebellar stimulation improves postural control, gait, and limb coordination in patients with ataxia [21], there is a dearth of research assessing the effects on cognitive and affective processing. It is well-known that degeneration of the cerebellum commonly leads to impairments in specific cognitive domains [3], but whether these functions can actually be enhanced by stimulation techniques requires further investigation. Furthermore, the precise mechanisms of action of (repeated sessions of) non-invasive cerebellar stimulation remain incompletely understood. Future research should be aimed at unravelling the underlying cellular processes, including possible changes in gene expression, protein synthesis, channel pump regulation, and modulation of receptors and/or neurotransmitters.

Many other questions regarding the relative effectiveness of different stimulation protocols remain unanswered. For instance, some recent studies have shown that iTBS over the dorsolateral prefrontal cortex is non-inferior to standard 10 Hz rTMS in treating depressive symptoms [182]. There is also promising evidence that cerebellar iTBS may be beneficial for improving gait and balance recovery in patients with middle cerebral artery and cerebellar strokes [183,184]. In addition, a new non-invasive brain stimulation technique called transcranial alternating current stimulation (tACS) has emerged, which can modulate cortical oscillations and entrain brain rhythms in specific frequencies [185]. Researchers have used cerebellar tACS at a frequency that matches the basal firing rate of Purkinje cells (i.e., 50 Hz) to modulate CBI and improve motor task performance in healthy individuals [186,187,188,189,190]. It remains to be determined whether these novel types of stimulation are also effective in treating cerebellar ataxias. Two tACS trials are currently underway in patients with ataxia (i.e., NCT05557786 and NCT05621200).

Finally, further work is warranted to define the optimal timing of follow-up stimulation sessions, to assess the feasibility of remotely supervised stimulation at home in larger groups of patients, and to evaluate if concurrent motor training interventions or pharmacologic therapies enhance the effects of non-invasive cerebellar stimulation. Demonstration of functional target engagement using imaging or neurophysiological biomarkers and a better understanding of interindividual differences in treatment response will be essential and may lead to the development of personalized stimulation protocols. Ongoing modeling studies in patients with different degrees of cerebellar atrophy will provide important further insights.

In summary, non-invasive (and perhaps also invasive) cerebellar stimulation may offer a promising approach for the treatment of ataxia, especially in view of the limited evidence-based pharmacological and non-pharmacological options currently available. However, further research is necessary before these techniques can be widely adopted in clinical practice.
